# Synergistic Stabilization of Zn Metal Anodes by 3D Carbon Frameworks with Multiple Ion Channels Loaded with Zincophilic BaTiO_3_ Nanoparticles

**DOI:** 10.1002/smsc.202400015

**Published:** 2024-04-09

**Authors:** Chuyi Li, Shengyang Jiang, Yang Li, Yongliang Li, Peixin Zhang, Chuanxin He, Lingna Sun, Hui Ying Yang

**Affiliations:** ^1^ College of Chemistry and Environmental Engineering Shenzhen University Shenzhen 518060 P. R. China; ^2^ Pillar of Engineering Product Development Singapore University of Technology and Design 8 Somapah Road Singapore 487372 Singapore

**Keywords:** anti‐corrosion, aqueous zinc‐ion batteries, electric field distribution, Zn anodes, Zn dendrites

## Abstract

Aqueous zinc‐ion batteries (AZIBs) suffer from rampant Zn dendrites growth, corrosion and sluggish transport kinetics, all of which have a serious impact on their performance and practical applications. Therefore, porous carbon nanofibers (BTO@PCNFs) loaded with BaTiO_3_ nanoparticles (BTO NPs) are constructed as a multifunctional interlayer for stabilizing the Zn anodes. Owing to the synergistic effect of BTO NPs and 3D porous carbon framework, this interlayer can achieve uniform electric field distribution, accelerate Zn^2+^ migration, and promote ion flux homogenization, thus leading to uniform Zn deposition. Meanwhile, the BTO@PCNFs interlayer demonstrates excellent anti‐corrosion effect, which can effectively prevent the side reactions. Benefiting from the synergistic effect of interlayers, the BTO@PCNFs multifunctional interlayers achieve a comprehensive optimization of the Zn anodes. The BTO@PCNFs‐Zn electrode exhibits lower nucleation overpotential (33.5 mV) at 0.5 mA cm^−2^. The Zn//Zn symmetrical cell with BTO@PCNFs interlayer exhibits ultra‐low overpotential (14 mV) and ultra‐long cycle life (5250 h at 0.5 mA cm^−2^). Even at high current density (30 mA cm^−2^), the BTO@PCNFs‐Zn electrode can be stably cycled for more than 830 h, achieving an ultra‐high cumulative capacity of 12 450 mAh cm^−2^. The multifunctional interlayers can provide a reference for the design of high‐performance Zn anodes.

## Graphical abstract

Zn anodes are inevitably subject to rampant Zn dendrites and corrosion. By covering the Zn foil with BTO@PCNFs membrane as a multifunctional interlayer, it can homogenize electric field distribution, regulate ion transport kinetics, uniform ion flux, prevent corrosion, and promote uniform Zn deposition, which realizes dendrite‐free Zn anode for long‐cycle‐life battery (5250 h at 0.5 mA cm^−2^ 0.5 mAh cm^−2^).

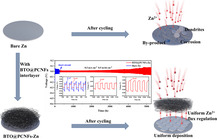

## 1 Introduction

Energy is becoming more and more in demand as society becomes more modernize.^[^ ^1^ ^]^ It is imperative to find clean energy and renewable resources because conventional fuels like coal and oil have finite reserves and degrade the ecosystemo.^[^ ^2^ ^]^ Among the current rechargeable battery technologies, the widely used lithium‐ion batteries (LIBs) are costly and the flammable electrolyte are also a major safety hazard, which severely hamper their further use in large‐scale applications.^[^ ^3^ ^]^ Compared to LIBs, aqueous zinc‐ion batteries (AZIBs) exhibit great potential for energy storage systems due to their safety, low cost, non‐toxicity, high theoretical capacity (820 mAh g^−1^, 5851 mAh cm^−3^), and low redox potential (−0.76 V vs standard hydrogen anodes).^[^ ^4^, ^5^, ^6^, ^7^, ^8^ ^]^ However, uncontrolled Zn dendrites growth, sluggish ion transport and corrosion seriously affect the practical application of AZIBs.^[^ ^9^, ^10^ ^]^

Rampant dendrites growth is a major “roadblock” limiting the practical application of AZIBs. It is widely believed that in the initial stripping/plating phase, Zn^2+^ tend to nucleate at lower energy sites; as the cycles continue, Zn protrusions gradually form, leading to initial Zn dendrites formation.^[^ ^9^, ^11^ ^]^ The “tip effect” of Zn dendrites exacerbates the distribution of uneven electric fields, facilitating two‐dimensional (2D) growth of dendrites.^[^ ^12^, ^13^ ^]^ As the exposed surface area increases, uncontrollable Zn dendrites may eventually puncture the separator, causing batteries short circuit.^[^ ^14^, ^15^ ^]^ Zn deposition is primarily influenced by the electric field distribution and ion concentration gradient.^[^ ^16^ ^]^ With the increment of current density, the polarization of Zn anodes rises correspondingly, leading to uneven Zn deposition.^[^ ^17^, ^18^ ^]^ At the same time, during the Zn deposition process, hydrogen evolution reactions (HER) occur, which can lead to an increase in local pH and accelerate corrosion and side reactions. As a result, Zn_4_SO_4_(OH)_6_·5H_2_O (ZHS) by‐products are formed, which create rough protrusions on the Zn anode. These poorly conducting by‐products increase interfacial impedance, lower ion transport rates, and aggravate Zn dendrites growth.^[^ ^14^, ^19^ ^]^

Numerous strategies have been employed to mitigate Zn dendrites formation and restrain corrosion reactions. These include the implementation of 3D conductive structures,^[^ ^20^, ^21^ ^]^ the use of zincophilic materials,^[^ ^22^, ^23^, ^24^ ^]^ electrolyte modification,^[^ ^25^, ^26^, ^27^ ^]^ and various other approaches. Based on a summary of previous studies,^[^ ^28^, ^29^, ^30^ ^]^ the ideal interface layer should fulfill the following conditions^[^ ^14^ ^]^: 1) proper zincophilicity and good wettability to the electrolyte for facilitating ion transport^[^ ^20^, ^31^ ^]^ 2) high specific surface area with abundant porous structure to homogeneous ion flux^[^ ^32^ ^]^; 3) good mechanical strength to keep the structure intact during cycling^[^ ^33^ ^]^ and 4d) physical and chemical stability to protect Zn anodes from corrosion.^[^ ^11^ ^]^

The dendrites formation, sluggish ion transport, and corrosion reactions in Zn anodes are not independent phenomena but rather mutually influenced and promoted, thus resulting in a vicious cycle.^[^ ^12^ ^]^ To overcome these challenges, it is imperative to create stable and efficient materials that can effectively regulate the Zn anodes. Previous studies have reported the use of carbon nanofiber (CNF) interlayers^[^ ^34^ ^]^ and BaTiO_3_^[^ ^35^, ^36^ ^]^ as coatings to enhance the performance of the Zn anodes. However, the binding energy of CNF to Zn is low and Zn tends to be deposited on the more conductive branches, leading to the growth of dendrites.^[^ ^37^ ^]^ The coating often peels off during cycling, causing damage to the battery.^[^ ^38^ ^]^ Introducing zincophilic materials into 3D carbon structures is an effective method for regulating Zn deposition.^[^ ^39^ ^]^ Furthermore, optimizing the pore size of a material is a sensible approach as narrow channels do not facilitate ion transport and materials with large pores will collapse during cycling due to low mechanical strength.^[^ ^38^, ^40^, ^41^, ^42^, ^43^ ^]^ Therefore, we constructed a 3D carbon framework with multiple ion channels loaded with zincophilic BTO NPs as an interlayer to synergistically stabilize the Zn anodes. It is worth noting that the previously reported BTO coating, BTO accounted for 83.3 wt%, 90 wt%, and so on.^[^ ^35^, ^36^, ^44^ ^]^ In our study, BTO accounts for only 3 wt%, which greatly reduces the amount of BTO compared to conventional coatings. In addition, the BTO@PCNFs film acts as an interlayer, which also avoids the use of binder. BTO@PCNFs membranes not only meet the above‐mentioned interfacial layer requirements, but also exhibit various advantages. The PCNFs membrane can be used as a physical barrier to avoid direct contact between the Zn anode and the electrolyte, thus inhibiting side reactions. Moreover, the formation of abundant nanopores in carbon nanofibers helps to achieve uniform ion flux, as well as the 3D carbon framework facilitates the reduction of local currents. The introduction of zincophilic BTO can accelerate the transport of Zn^2+^ and improve the kinetics. The BTO is polarized under the applied electric field of the cycling process,^[^ ^45^, ^46^, ^47^, ^48^ ^]^ and the generation of directional electric field can further regulate the electric field density distribution.^[^ ^36^ ^]^ It is worth noting that BTO@PCNFs membranes with negative surface charges not only accelerate Zn^2+^ transport, but also shield anions and prevent corrosion of Zn anodes. Based on the synergistic regulation of BTO NPs and PCNFs membranes, Zn anodes modified with BTO@PCNFs interlayers have outstanding electrochemical performance. The Zn//Zn symmetric cells with BTO@PCNFs interlayers exhibited excellent cycling stability (5250 h, at 0.5 mA cm^−2^; 830 h, at 30 mA cm^−2^). Additionally, BTO@PCNFs‐Zn//α‐MnO_2_ full cells and BTO@PCNFs‐Zn//VO_2_ full cells both show excellent stability. The exceptional performance demonstrated by the BTO@PCNFs interlayer can provide a valuable reference for achieving high‐performance AZIBs.

## 2 Results and Discussion

**Figure 1:**
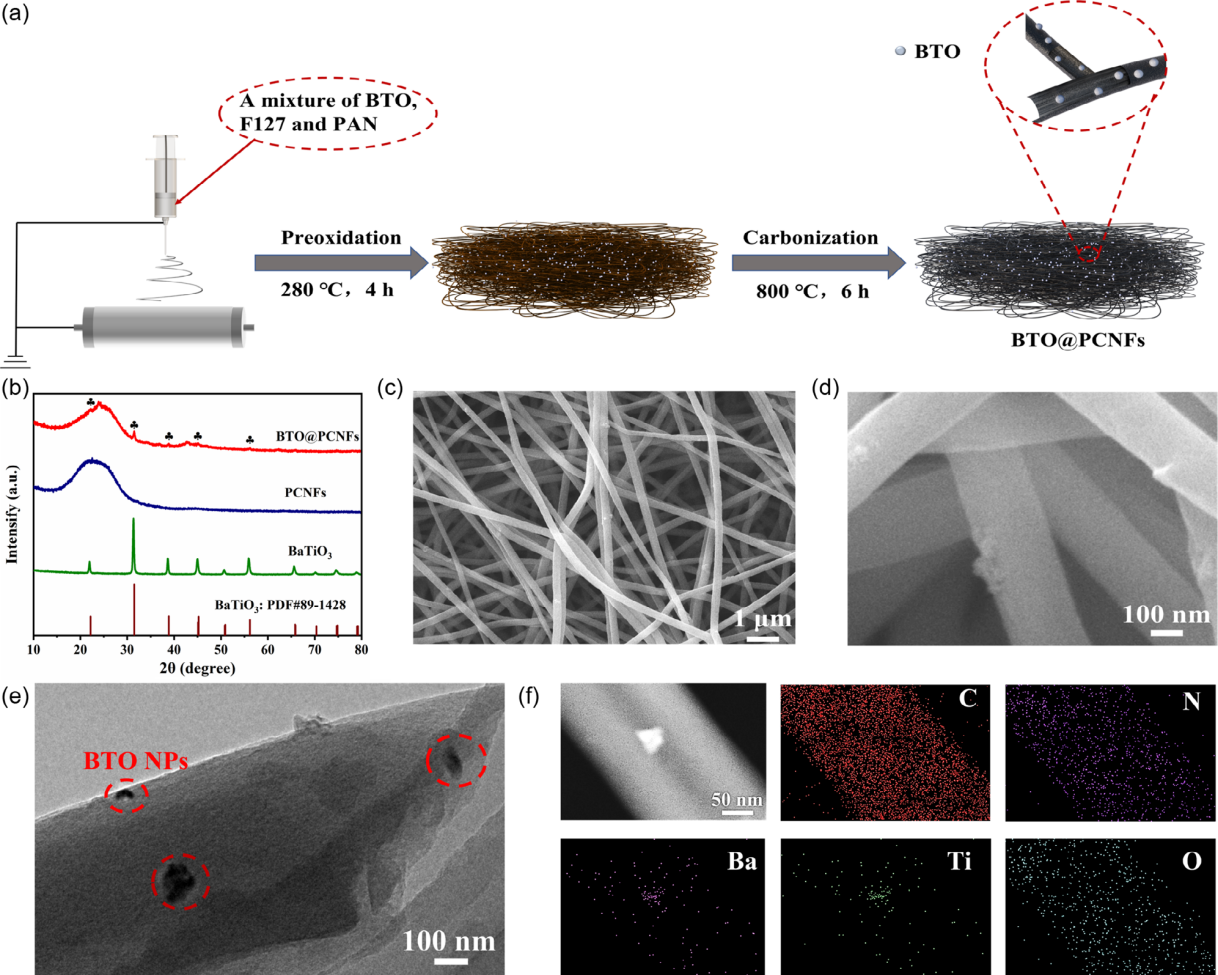
a) Schematic illustration for BTO@PCNFs membrane preparation process. b) XRD patterns of BTO, PCNFs and BTO@PCNFs membranes. c,d) FESEM images of BTO@PCNFs membrane. e) TEM image of BTO@PCNFs membrane. f) TEM image of BTO@PCNFs membrane and corresponding elemental mappings.

The schematic diagram of BTO@PCNFs membrane synthesis is shown in **Figure** **1**a. Polyacryonitrile (PAN), BTO NPs and triblock copolymer F127 were added to N, N‐dimethylformamide solvent (DMF) and stirred to form a spinning solution.^[^ ^49^ ^]^ Subsequently, the BTO@PCNFs membrane with multiple nanopores were obtained by pre‐oxidation and carbonization. As a polymeric nonionic surfactant, F127 can enhance the dispersion and distribution of BTO NPs in carbon nanofibers. Additionally, it can serve as a soft template during the carbonization process, resulting in the formation of porous structures in the carbon nanofibers.^[^ ^50^, ^51^, ^52^, ^53^ ^]^ The characterization of BTO@PCNFs using TEM shows that they contain a significant number of nanopores (Figure S1, Supporting Information). The specific surface area and pore size distribution of BTO@PCNFs were measured by nitrogen adsorption‐desorption tests (Figure S2, Supporting Information), which showed that the Brunauer–Emmett–Teller specific surface area was 169.57 m^2^ g^−1^ and the pore size was mainly distributed in the range of 1–3 nm. Nanopores were also confirmed to facilitate electrolyte transport and achieve uniform ion flux, thus kinetically improving the electrochemical performance of the Zn anodes.^[^ ^43^, ^54^ ^]^ Additionally, the larger surface area provided by the nanopores promotes uniform local currents and lower overpotentials.^[^ ^55^, ^56^ ^]^ The XRD pattern proved the successful synthesis of BTO@PCNFs (Figure 1b). The strong peak of BTO (101) corresponded to the standard card (PDF#89‐1428) and some small peaks intensity were not obvious due to the low addition of BTO (Figure S3, Supporting Information). To determine the weight percentage of BTO in the BTO@PCNFs membrane, the BTO content was approximately only 3 wt% by comparing the thermogravimetric (TG) results of PCNFs and BTO@PCNFs (Figure S4, Supporting Information). Besides, the synthesized BTO@PCNFs membranes have a certain flexibility and great mechanical strength (Figure S5a,c, Supporting Information). The tensile strength of the CNF interlayer reported in the literature was about 0.25 MPa and the thickness was about 66 μm.^[^ ^34^ ^]^ The tensile strength of the BTO@PCNFS membrane synthesized in this work is 1.69 MPa, which is 6.76 times that of the reported CNF interlayer. Simultaneously, the BTO@PCNFs membrane with a diameter of 16 mm underwent folding and unfolding at various angles. Remarkably, upon removal of the external force, the folded area of the BTO@PCNFs membrane exhibited the ability to revert back to its original state, thus confirming its inherent flexibility (Figure S5c, Supporting Information). Whereas designing carbon nanofibers of a certain thickness (78.7 μm) forms a 3D structure that can reduce local currents as well as provide more nucleation sites (Figure S5b, Supporting Information).^[^ ^57^ ^]^ The design of a 3D porous carbon skeleton with high mechanical strength can prevent the anode structure from collapsing during battery cycling. Under FESEM, the BTO@PCNFs appeared uniformly dispersed (Figure 1c). By adjusting the magnification, the BTO NPs could be observed scattered within the carbon nanofibers (Figure 1d). TEM image clearly showed BTO nanoparticles loaded on carbon nanofibers. (Figure 1e). The presence of C, N, Ba, Ti, O by TEM‐EDS analysis (Figure 1f) also corroborated the successful synthesis of BTO@PCNFs, which was consistent with XRD pattern.

**Figure 2:**
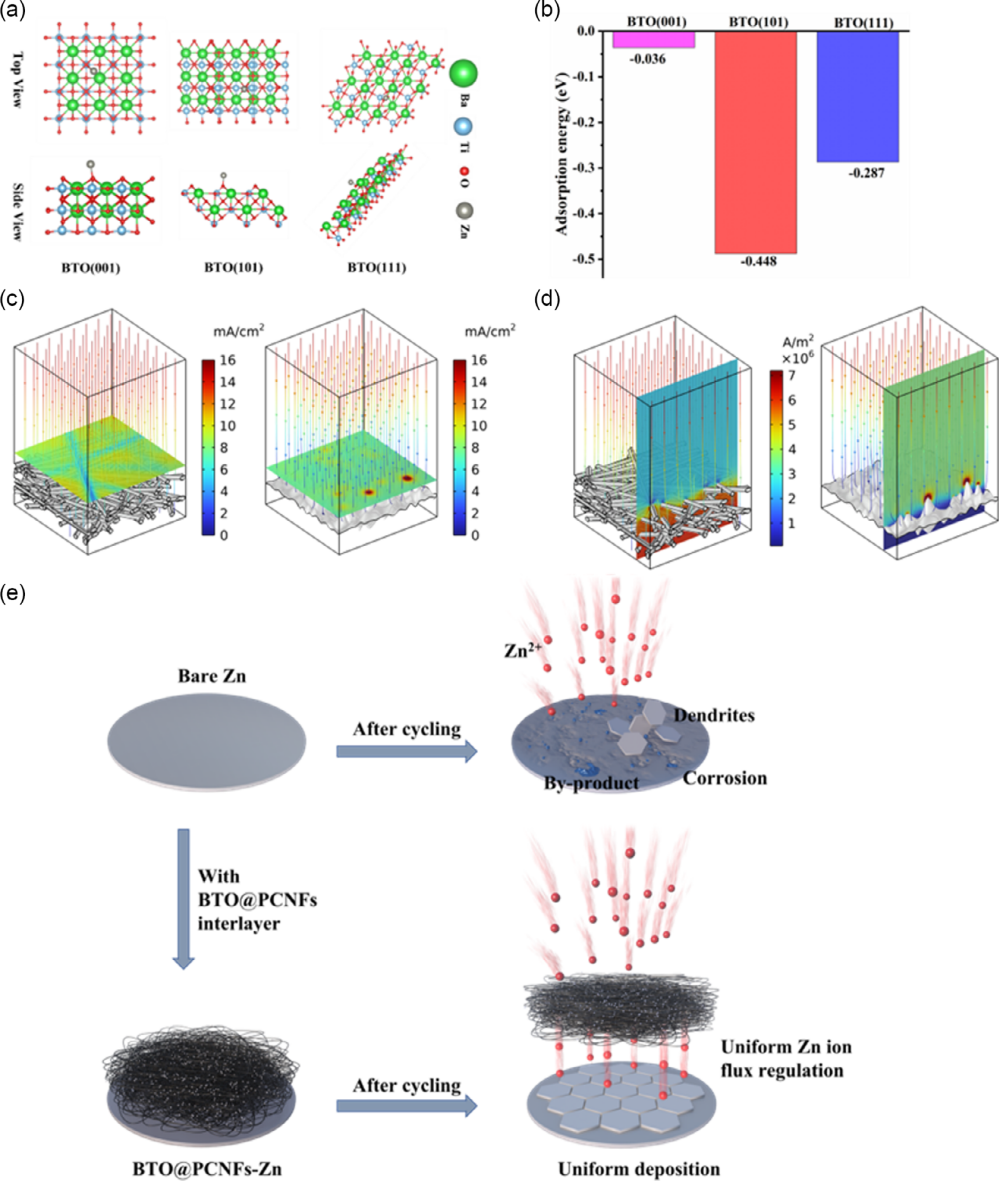
a) Schematic diagram of the geometric configuration of the (001), (101), and (111) faces of the BTO bonded to the Zn atom. b) Binding energy of Zn atoms to different adsorption sites of BTO. c) Current density distribution diagram of BTO@PCNFs membrane (left), bare Zn (right). d) Cross‐sectional diagram of current density distribution of BTO@PCNFs membrane (left), bare Zn (right). e) Schematic illustration of Zn stripping/plating behavior with/without BTO@PCNFs membrane.

BTO, as a material with high dielectric coefficient and piezoelectric properties, can be induced to polarize under the influence of external electric field or external force, forming an internal electric field.^[^ ^58^ ^]^ We selected BTO NPs of non‐centrosymmetric tetragonal crystal system and loaded them onto carbon nanofibers by electrostatic spinning technique as the main active component in the interlayer for modulating Zn deposition. During the cycling process, the Ti^4+^ cation may deviate from the [TiO_6_] center and shift relative to the negatively charged oxygen under the influence of the external electric field, resulting in a polarization parallel to the electric field that can direct the ordered migration of Zn^2+^.^[^ ^36^, ^47^, ^48^, ^59^ ^]^ Zincophilic materials facilitate accelerated Zn^2+^ transport as well as modulate Zn deposition,^[^ ^60^ ^]^ and we have explored the interaction of BTO with Zn atoms from a theoretical analysis by using density functional theory (DFT) calculations (**Figure** **2**a). In this experiment, we studied three crystal faces of BTO: (001), (101), and (111), as displayed in Figure 2b. Our results showed that the adsorption energies of Zn atoms on these faces were −0.036, −0.448, and −0.287 eV, respectively. The attraction of the BTO (101) crystal face to the Zn atoms is particularly prominent and stronger than the other two crystal faces. The appropriate affinity between BTO and Zn enables BTO to serve as zincophilic sites, which is advantageous for promoting ion transport, lowering the nucleation potential barrier, and regulating Zn deposition. The electric field is the driving force for ion transport,^[^ ^61^ ^]^ and to investigate the effect of BTO@PCNFs membrane on the electric field, we simulated the local current density distribution of BTO@PCNFs and bare Zn by COMSOL multiphysics finite element method (FEM). Since the surface of commercially available Zn foil does not reach a smooth and flat surface, the surface charge is not uniformly distributed (Figure 2c right). In the initial deposition stage, Zn^2+^ tend to nucleate at lower energy sites and gradually aggregate to form protrusions, and the local electric field formed by the “tip effect” further attracts Zn^2+^ to form the final Zn dendrites.^[^ ^62^ ^]^ The cross‐sectional view of the bare Zn model (Figure 2d right) shows that a local current is formed in the protrusions of the zinc foil, which induces Zn deposition and thus leads to Zn dendrites growth. To simulate the current density distribution of BTO@PCNFs, BTO were incorporated into carbon nanofibers (Figure 2c left, 2d left). The zincophilic BTO together with the conductive 3D carbon nanofiber skeleton can homogenize the current density distribution to inhibit Zn dendrites growth. In Figure 2e, the inherent rough surface of the bare Zn electrode causes local current to rise during the cycling process, thus evolving into uncontrollable dendrite growth, and the fragile dendrites easily fall off from the electrode to form “dead Zn”;^[^ ^63^ ^]^ the electrolyte and direct contact with the electrode always inevitably cause corrosion and by‐products that seriously affect Zn anode reversibility.^[^ ^64^ ^]^ The side reactions and Zn dendrite growth are interacting with each other, so it is necessary to develop a high‐performance Zn anode. In our study, BTO@PCNFs membrane was covered on Zn foil as an interlayer to protect the Zn anodes and to regulate the uniform Zn deposition. The BTO@PCNFs interlayer can achieve synergistic effects. Zincophilic BTO can adsorb Zn^2+^, accelerate ion transport and lower the nucleation potential. The porous carbon fibers with a 3D structure can provide more nucleation sites and uniform ion flux. Meanwhile, the BTO@PCNFs membrane can not only uniform the current density but also effectively suppress the corrosion reactions. The combination of BTO and 3D porous carbon framework exhibits complementary effects and effectively inhibits the Zn dendrites growth, resulting in a high‐performance Zn anode.

**Figure 3:**
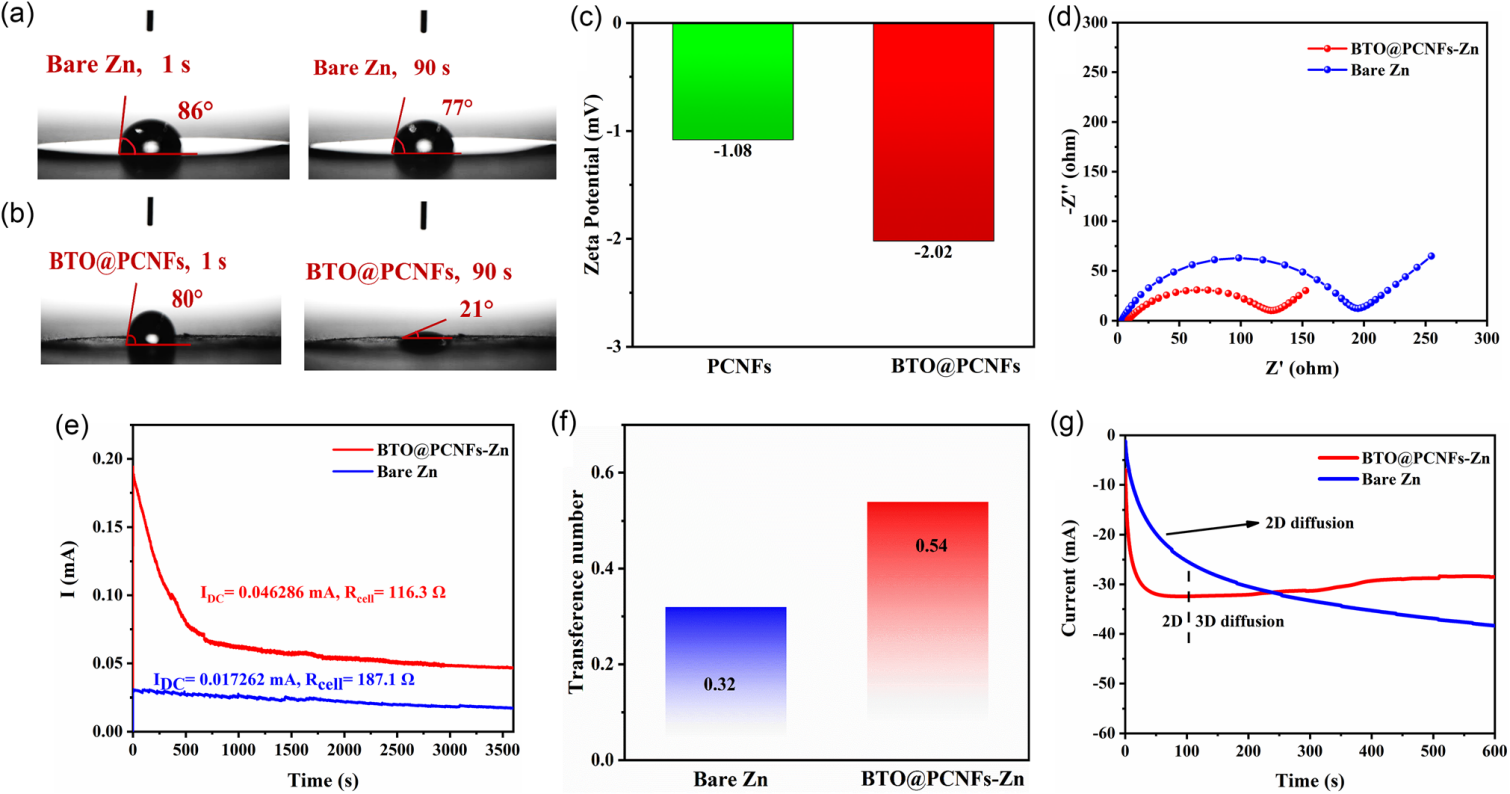
a) Contact angles of bare Zn. b) Contact angles of the BTO@PCNFs membrane. c) Zeta potential tests of PCNFs membrane and BTO@PCNFs membrane. d) Nyquist plots of the symmetric cells of bare Zn and BTO@PCNFs‐Zn. e) Time‐dependence response of potentiostatic DC polarization for Zn//Zn symmetric cells. f) Calculated transfer number of Zn^2+^ (t_Zn2+_). g) CA of bare Zn and BTO@PCNFs‐Zn at −150 mV overpotential.

Sluggish ion transport will increase the concentration polarization and have a significant impact on the stripping/plating of Zn^2+^. Improving ion transport kinetics can facilitate the elimination of cumulative effects caused by the stripping/plating process and promote uniform Zn deposition.^[^ ^65^ ^]^ The high wettability of the electrolyte facilitates the reduction of the free energy between the Zn anode and the electrolyte, thus promoting the interfacial ion transfer.^[^ ^66^ ^]^ During the contact angle tests with electrolyte (2 m ZnSO_4_), the initial contact angle of bare Zn was measured to be 86°, which decreased to 77° at the 90th second (**Figure** **3**a). In contrast, the BTO@PCNFs membrane had an initial contact angle of 80°, but it wetted rapidly, and the contact angle decreased to 21° at the 90th second (Figure 3b). The contact angle decreased quickly, indicating that BTO@PCNFs membrane exhibited strong attraction to the electrolyte. This is beneficial for facilitating rapid ion transfer and reducing interfacial ion transfer resistance. To investigate the reason for the accelerated ion transport in BTO@PCNFs membrane, zeta potential test was used to examine the surface charge of both the PCNFs membrane and the BTO@PCNFs membrane (Figure 3c). The results showed that the average potential of the PCNFs membrane was −1.08 mV, while the average potential of the BTO@PCNFs membrane was −2.02 mV. They both exhibited negative potential. In contrast, the BTO@PCNFs membrane have lower potential, indicating that the BTO@PCNFs membrane can better attract Zn ions. From the analysis of the electrochemical impedance spectroscopy (EIS) of the symmetric cell (Figure 3d), it was known that the R_ct_ of the BTO@PCNFs‐Zn electrode (116.3 Ω) was significantly lower than that of the bare Zn electrode (187.1 Ω), indicating that the BTO@PCNFs interlayer could promote charge transfer. To better demonstrate the function of the BTO@PCNFs interlayer in ion transport, potentiostatic direct‐current (DC) polarization tests were performed to measure the Zn^2+^ transference number (t_Zn2+_) of the symmetric cell (Figure 3e–f). The t_Zn2+_ of the symmetric cell with bare Zn is 0.32 and increases to 0.54 for the symmetric cell with the addition of the BTO@PCNFs interlayer. To investigate the Zn deposition behavior of the Zn anode, a constant voltage of −150 mV was applied to the symmetric cell using chronoamperometry (CA), and the nucleation process and interfacial changes were investigated by the variation of current versus time.^[^ ^29^ ^]^ For the bare Zn electrode (Figure 3g), the current density increased continuously over 600 s, indicating that the deposited Zn^2+^ diffused along the surface 2D and the aggregated Zn^2+^ tended to form rampant Zn dendrites. In contrast, the BTO@PCNFs‐Zn electrode exhibited a stable current density after 102 s, indicating that the BTO@PCNFs interlayer was effective in inhibiting the 2D diffusion of Zn^2+^. In summary, the BTO@PCNFs interlayer can accelerate ion transport, resulting in uniform ion flux through the abundant nanochannels. Additionally, the uniform distribution of the electric field promotes stable Zn stripping/plating and the 3D diffusion of Zn^2+^ on the Zn anode surface.

**Figure 4:**
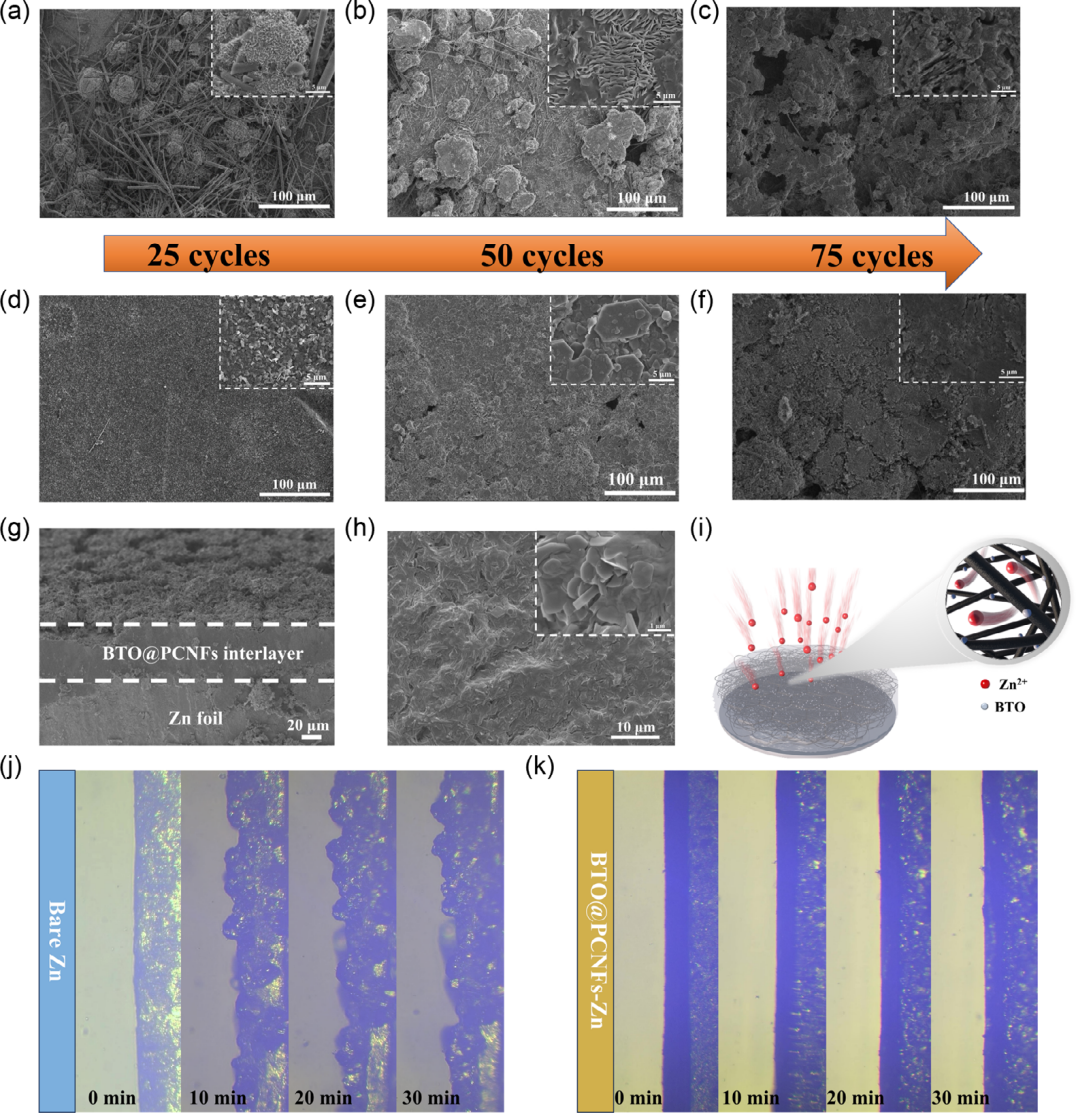
Images of Zn foil surface morphology after cycling at 5 mA cm^−2^, 5 mAh cm^−2^: a) Bare Zn electrode after 25 cycles; b) Bare Zn electrode after 50 cycles; c) Bare Zn electrode after 75 cycles; d) BTO@PCNFs‐Zn electrode after 25 cycles; e) BTO@PCNFs‐Zn electrode after 50 cycles; f) BTO@PCNFs‐Zn electrode after 75 cycles.(Observation after removal of BTO@PCNFs membrane) g) Cross‐sectional FESEM images of BTO@PCNFs‐Zn electrode after 75 cycles at 5 mA cm^−2^, 5 mAh cm^−2^. h) BTO@PCNFs membrane after 75 cycles at 5 mA cm^−2^, 5 mAh cm^−2^. i) Schematic of BTO NPs attracting Zn^2+^ and Zn deposition. j,k) Zn deposition of bare Zn electrode and BTO@PCNFs‐Zn electrode observed using in situ optical microscopy at 20 mA cm^−2^.

To investigate the effect of the BTO@PCNFs interlayer in regulating Zn deposition, the morphology of Zn anodes after cycling were observed by assembling symmetric cells. Symmetric cells cycled at 5 mA cm^−2^, 5 mAh cm^−2^ for 25 cycles, 50 cycles and 75 cycles were respectively chosen to observe the evolution of the deposited morphology of the Zn anodes. Through SEM images, it could be observed that bare Zn tended to aggregate and deposit Zn^2+^ into a Zn cluster after 25 cycles of cycling (**Figure** **4**a). Due to the “tip effect”, Zn^2+^ tended to grow at the initial Zn protrusions, gradually forming rougher Zn dendrites (Figure 4b). As the cycle time increased, the Zn deposition gathered to form a more fragile porous Zn (Figure 4c), and the increased exposure area led to more severe corrosion of the Zn anode, which severely destabilized the Zn anode. In contrast, Zn anode with BTO@PCNFs interlayer underwent reversible stripping/plating at 5 mA cm^−2^, 5 mAh cm^−2^. The Zn ions were deposited uniformly on the Zn foil through the modulation of the interlayer. After 25 cycles of BTO@PCNFs‐Zn electrode (Figure 4d), the Zn foil surface was flat and dense, and no by‐products were found. Even after 50 cycles, 75 cycles (Figure 4e–f), the Zn deposition remained flat and dense. By XRD testing of Zn anodes after cycles, bare Zn had clear Zn_4_SO_4_(OH)_6_·5H_2_O diffraction peaks at 8°, but no by‐product peaks were visible for BTO@PCNFs‐Zn (Figure S6, Supporting Information). It indicated that the BTO@PCNFs interlayers could effectively inhibit the generation of by‐products during the cycling process, which was conducive to promoting uniform Zn deposition. Interestingly, the BTO@PCNFs membrane adhered closely^[^ ^67^ ^]^ to the Zn foil after 75 cycles at 5 mA cm^−2^, 5 mAh cm^−2^ (Figure 4g). Figure 4h displays FESEM images of the BTO@PCNFs interlayer after cycling, showing carbon fiber skeleton is distributed with uniform Zn deposition. Additionally, FESEM‐EDS images (Figure S7, Supporting Information) confirm the presence of elements C, N, O, Ba, Ti, and Zn, with a consistent distribution of Zn elements observed. This further supports the conclusion that Zn is uniformly deposited on the BTO@PCNFs interlayer. As illustrated in Figure 4i, the zincophilic BTO NPs can attract Zn^2+^ and lowering the formation barrier. This allows the zinc ions to be deposited in the porous 3D carbon framework. Following cycling, the Zn foil becomes firmly attached to the BTO@PCNFs membrane, resulting in a shorter ion transport path. Additionally, the rapid ion transport aids in the uniform Zn deposition.^[^ ^34^ ^]^ To further explore the modulating effect of BTO@PCNFs membrane on Zn deposition, in situ optical microscopy was used to observe the Zn deposition process of bare Zn electrode and BTO@PCNFs‐Zn electrode in real time. Under high current density conditions of 20 mA cm^−2^, bare Zn electrodes first formed obvious protrusions and with continuous growth formed Zn dendrites, which can be visualized as obvious little protrusions after 10 min (Figure 4j). After 20 and 30 min, the previous Zn dendrites were still growing uncontrollably. An interesting phenomenon was that the color of the electrolyte became darker as the plating time increased, which is presumably due to the uncontrolled growth of Zn dendrites at high current density and the formation of by‐products and “dead Zn” during the deposition process.^[^ ^68^ ^]^ For the BTO@PCNFs‐Zn electrode, there were no visible protrusions were observed even after 30 min of plating (Figure 4k). In addition, the BTO@PCNFs interlayer adhered more and more closely to Zn foil, which was consistent with the above finding (Figure 4g).

**Figure 5:**
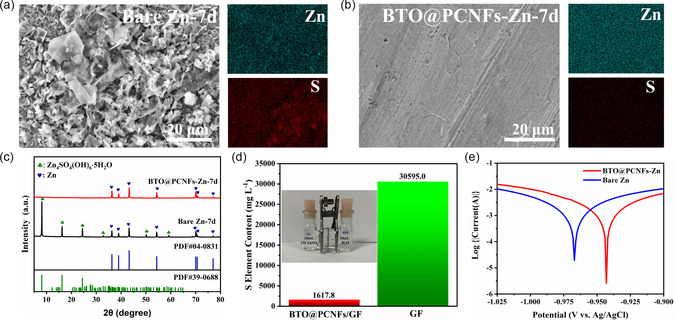
a) Corresponding EDS mapping of Zn, S elements of bare Zn after 7 days of immersion in 2 m ZnSO_4_ electrolyte. b) Corresponding EDS mapping of Zn, S elements of BTO@PCNFs‐Zn after 7 days of immersion in 2 m ZnSO_4_ electrolyte. c) XRD patterns of Bare Zn and BTO@PCNFs‐Zn after 7 days of immersion in 2 m ZnSO_4_ electrolyte. (Test after removal of BTO@PCNFs membrane) d) SO_4_^2−^ permeability of BTO@PCNFs/GF and GF membranes. e) Linear polarization curves showing the corrosion on Bare Zn electrode and the BTO@PCNFs‐Zn electrode in 2 m ZnSO_4_ electrolyte.

It is known that the instability of Zn anode is also due to Zn corrosion. The Zn corrosion generates alkaline zinc sulfate by‐products (Zn_4_SO_4_(OH)_6_·5H_2_O) that cause a decrease in Zn utilization, and the by‐products covering the surface of the Zn anodes hinder ion transport.^[^ ^69^ ^]^ To investigate the protective effect of BTO@PCNFs membrane on Zn foil, Bare Zn and protected Zn foil (BTO@PCNFs‐Zn) were immersed in 2 m ZnSO_4_ electrolyte for 7 days. The surface morphology of the immersed Bare Zn and Zn foil with BTO@PCNFs membrane removed was observed by FESEM, and the surface material composition of Zn foil was identified by XRD tests. In **Figure** **5**a, the surface of Bare Zn is very rough, and fluffy by‐products cover the Zn foil, indicating that the unprotected Bare Zn is severely corroded. During the cycling process, the uneven Zn anode surface affects the current density distribution and increases the electrode polarization, which can lead to Zn dendrites growth.^[^ ^70^ ^]^ In contrast, the surface of the protected Zn foil (BTO@PCNFs‐Zn) is flat (Figure 5b). As seen from the EDS mapping (Figure 5a,b), the sulfur (S) element almost covers the Bare Zn surface, while the protected Zn foil (BTO@PCNFs‐Zn) has a weak S element signal (Figure S8, Supporting Information), indicating that the unprotected Bare Zn surface generated a large number of sulfur‐containing by‐products. In the XRD patterns of Figure 5c, the intensity of the Zn_4_SO_4_(OH)_6_·5H_2_O diffraction peak of the Bare Zn soaked for 7 days was relatively strong, while almost no Zn_4_SO_4_(OH)_6_·5H_2_O diffraction peak was detected for the protected Zn foil (BTO@PCNFs‐Zn). This corroborates the excellent corrosion resistance of the BTO@PCNFs membrane. The ion permeability of BTO@PCNFs membranes was detected by H‐Cells (Figure 5d) with 10 mL of 2 m ZnSO_4_ electrolyte on one side and 10 mL of deionized water on the other side and left for one hour, and the deionized water was collected for analysis by inductively coupled plasma optical emission spectrometer (ICP‐OES). Under the same conditions, the S element content was significantly less with BTO@PCNFs/GF membrane than with glass fiber (GF) membrane. This also proves that BTO@PCNFs membranes can shield anions (SO_4_^2−^) and restrain the generation of by‐products. The corrosion resistance of the BTO@PCNFs membrane was investigated by line polarization tests (Figure 5e). In the 2 m ZnSO_4_ electrolyte, the BTO@PCNFs‐Zn had a higher corrosion potential (−0.943 V) compared to that of Bare Zn (−0.967 V), indicating a lower tendency of corrosion reaction. Furthermore, the decrease in corrosion current indicated that the corrosion efficiency was reduced, and the Zn corrosion reactions were inhibited.

**Figure 6:**
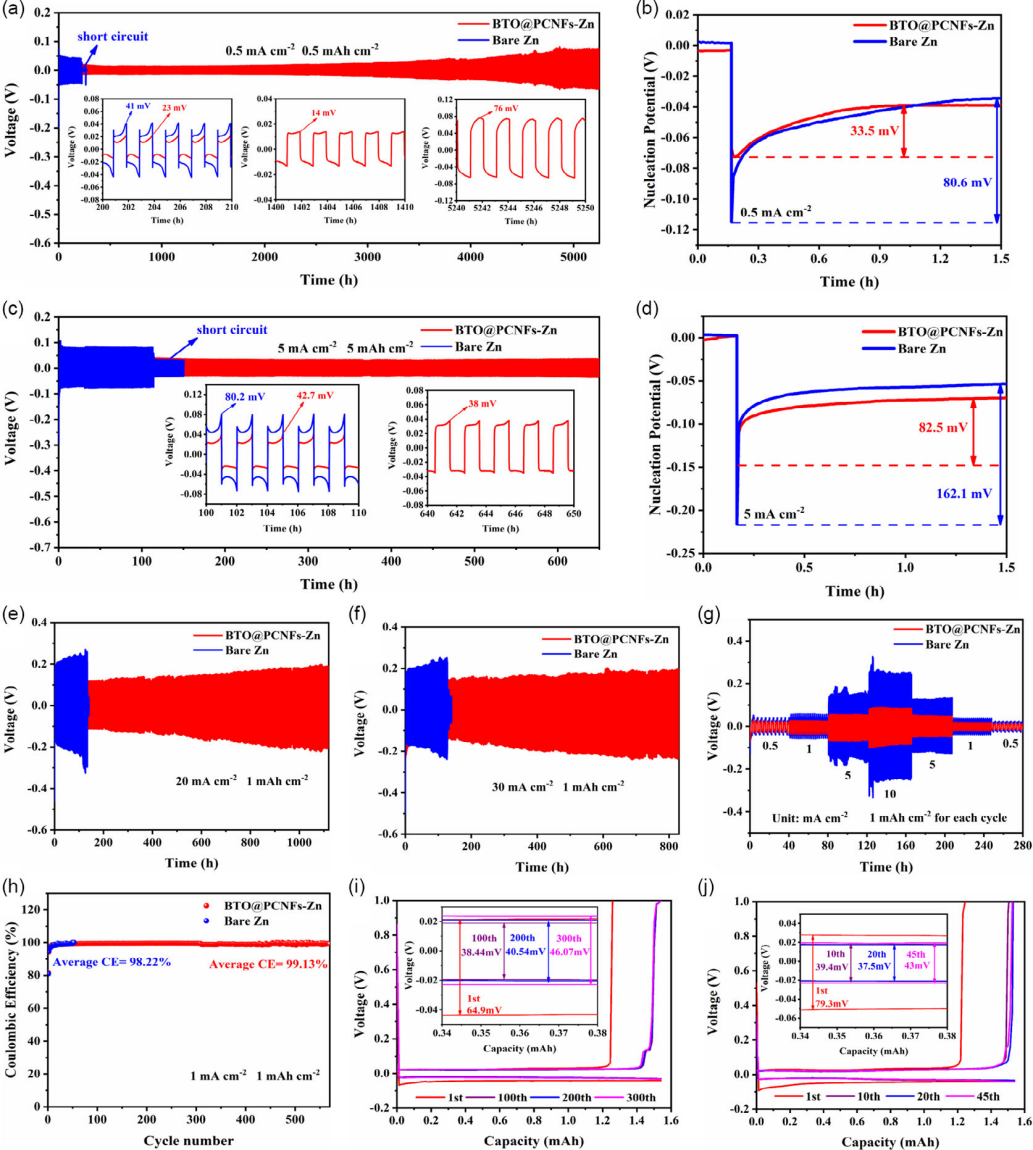
Cycling performance of symmetric cells and corresponding nucleation overpotential at the condition of: a,b) 0.5 mA cm^−2^, 0.5 mAh cm^−2^; c,d) 5 mA cm^−2^, 5 mAh cm^−2^; e) Cycling performance of symmetric cells at 20 mA cm^−2^, 1 mAh cm^−2^; f) Cycling performance of symmetric cells at 30 mA cm^−2^, 1 mAh cm^−2^. g) Rate performance of the symmetric cells at different current densities. h) Coulombic efficiency of Zn//Cu asymmetric cells at 1 mA cm^−2^, 1 mAh cm^−2^. i,j) Galvanostatic charge/discharge profiles of BTO@PCNFs‐Zn//Cu asymmetric cell and Bare Zn//Cu at 1 mA cm^−2^, 1 mAh cm^−2^.

To study the modulating effect of BTO@PCNFs interlayer on Zn stripping/plating, its stability was estimated by assembling Zn//Zn symmetric cells. This study was based on a porous 3D carbon framework as an interlayer, and the amount of BTO introduced was also critical to further improve the electrochemical performance. Unlike conventional BTO coatings, we speculated that a small amount of BTO addition could improve batteries performance. Excessive addition of BaTiO_3_ nanoparticles can lead to aggregation, which will affect the electrode performance.^[^ ^71^ ^]^ To determine the optimal amount of BTO to use, an experiment was conducted in which 10, 30, and 50 mg of BTO (referred to as BTO‐10@PCNFs, BTO‐30@PCNFs, and BTO‐50@PCNFs, respectively) were added. As a control group, those without the addition of BTO were recorded as PCNFs. The BTO optimal addition amount was determine by comparing the cycle performance of symmetric cells. The cycling stability of the BTO‐30@PCNFs‐Zn electrode was better than that of the BTO‐10@PCNFs‐Zn and BTO‐50@PCNFs‐Zn electrodes (0.5 mA cm^−2^, 0.5 mAh cm^−2^; 5 mA cm^−2^, 5 mAh cm^−2^) and much better than that of the PCNFs‐Zn electrode (Figure S9a,b, Supporting Information). For the convenience of description, BTO‐30@PCNFs‐Zn is denoted as BTO@PCNFs‐Zn. It is noteworthy that the BTO@PCNFs‐Zn electrode exhibits excellent reversibility, as it could be stably cycled for over 5250 h at 0.5 mA cm^−2^ and 0.5 mAh cm^−2^ (**Figure** **6**a). In contrast, the Bare Zn electrode exhibited a higher overpotential (41 mV) as well as a short cycle life (217 h). Surprisingly, the BTO@PCNFs‐Zn electrode started cycling at a low overpotential (23 mV) for some time, followed by a very low overpotential (14 mV) for a long time. The ultra‐low polarization voltage of the Zn//Zn symmetric cell was attributed to the BTO@PCNFs interlayer which largely inhibited corrosion side reactions and improved ion transport kinetics. Additionally, at 0.5 mA cm^−2^ (Figure 6b), the nucleation overpotential of the BTO@PCNFs‐Zn electrode (33.5 mV) is much lower than that of the bare zinc electrode (80.6 mV). Even under different current density conditions, the nucleation overpotential of the BTO@PCNFs‐Zn electrodes increased with the increase of current density, but they were consistently lower than that of the bare Zn electrodes, indicating that the low nucleation overpotential was attributed to the modulation of the BTO@PCNFs interlayer (Figure 6d and S9e–g, Supporting Information). This may be because the zincophilic BTO can attract Zn^2+^, and the 3D porous carbon framework can absorb more electrolyte ions, which can provide more nucleation sites.^[^ ^72^ ^]^ The BTO and PCNFs synergistically reduce the Zn nucleation barriers, thus forming smaller initial Zn nucleation and inhibiting the formation of Zn dendrites.^[^ ^55^, ^73^ ^]^ At 5 mA cm^−2^, 5 mAh cm^−2^ (Figure 6c), the bare Zn electrode had large voltage fluctuations (80.2 mV) and started to short circuit after 114 h of cycling; whereas the BTO@PCNFs‐Zn electrode was able to achieve stable Zn stripping/plating and exhibited a low overpotential (40.7 mV) in the early stage, followed by stable cycling at a overpotential of 37 mV with a cycle life of over 650 h. However, at high current densities, the growth of Zn dendrites became even more rampant, which led to the rapid short‐circuiting of the cell.^[^ ^17^ ^]^ To further investigate the ability of the BTO@PCNFs interlayer in regulating the Zn anode, high‐current galvanostatic charge/discharge cycle tests were performed. At 10 mA cm^−2^, 10 mAh cm^−2^ (Figure S9c, Supporting Information), the Bare Zn electrode had large voltage fluctuations with overpotential up to 300 mV and started short‐circuiting after 110 h, while the BTO@PCNFs‐Zn electrode could cycle with stable overvoltage for more than 310 h. Remarkably, the BTO@PCNFs‐Zn electrode maintained an ultra‐long cycle life even at higher current density conditions. At 20 mA cm^−2^, 1 mAh cm^−2^, the BTO@PCNFs‐Zn electrode could achieve stable stripping/plating for more than 1120 h, and even at 30 mA cm^−2^, 1 mAh cm^−2^, the cycle life exceeded 830 h. To investigate the effect of BTO@PCNFs‐Zn electrodes for practical applications, symmetric cells were assembled under 49% depth of discharge (DOD) and high current density conditions (20 mA cm^−2^, 20 mAh cm^−2^) selected in this work. Bare Zn electrodes exhibit high polarization voltages followed by short circuits after 60 h. BTO@PCNFs can still cycle stably for 250 h at high DOD and high current density (Figure 6d). As shown in Figure 6g, the overpotential of the BTO@PCNFs‐Zn symmetric cell was significantly lower than that of the Bare Zn symmetric cell at different current densities. When transitioning from low current densities of 0.5 mA cm^−2^ and 1 mA cm^−2^ to higher current densities of 5 mA cm^−2^, the bare Zn cell started to undergo strong voltage fluctuations, which were more intense at 10 mA cm^−2^ current density. This is because at high current densities, this leads to rapid growth of Zn dendrites. By gradually increasing the current density to 10 mA cm^−2^ and then returning to the starting current density of 0.5 mA cm^−2^, the BTO@PCNFs‐Zn cell still maintained normal cycling, indicating that the BTO@PCNFs‐Zn cell had superior cycling performance. Notably, the BTO@PCNFs‐Zn electrode has a maximum cumulative plating capacity of 12 450 mAh cm^−2^ (30 mA cm^−2^ 1 mAh cm^−2^), which is 4.15 times that of the CNF‐Zn^[^ ^34^ ^]^ electrode and 1.92 times that of the Corona‐poled Zn@BaTiO_3_^[^ ^35^ ^]^ electrode (Table S1, Supporting Information). The BTO@PCNFs‐Zn electrodes also exhibit outstanding performance compared to the performance of Zn‐based symmetric batteries reported in journals in the last 2 years (Table S2, Supporting Information). To further elucidate the role of BTO@PCNFs interlayer on Zn stripping/plating reversibility, coulombic efficiency (CE) of Zn//Cu asymmetric cells was investigated. Bare Zn//Cu cell started short‐circuiting at the 50th cycle with an average CE of only 98.22%, while BTO@PCNFs‐Zn//Cu cell was able to cycle steadily for 570 cycles with an average CE of 99.13% (Figure 6h and S10a,b, Supporting Information), demonstrating the great stripping/plating reversibility of BTO@PCNFs‐Zn electrode. Meanwhile, the BTO@PCNFs‐Zn electrode had a lower initial overpotential (64.9 mV), and the overpotential after 100 cycles is 38.44 mV (Figure 6i), while the Bare Zn electrode reaches 43 mV after 45 cycles (Figure 6j), indicating that the Zn//Cu asymmetric cells with the addition of the BTO@PCNFs interlayer can improve the electrochemical performance.

**Figure 7:**
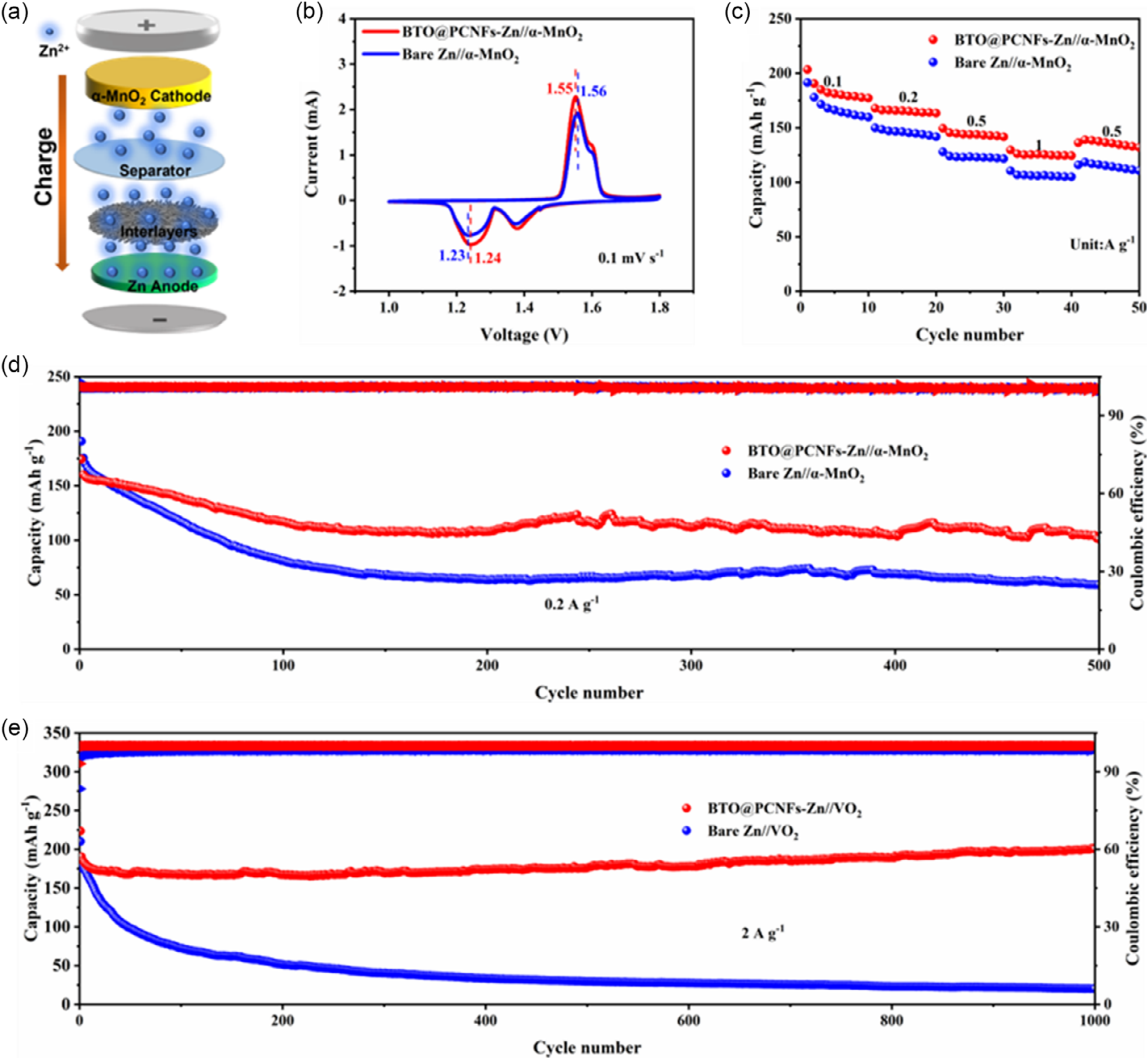
a) Schematic diagram of the full cell of BTO@PCNFs‐Zn//α‐MnO_2_. b) CV curves of the second cycle of Zn//α‐MnO_2_ full cell. c) Rate performance of Zn//α‐MnO_2_ full cells. d) Cycling performance BTO@PCNFs‐Zn//α‐MnO_2_ full cells. e) Cycling performance of Zn//VO_2_ full cells.

To further investigate the feasibility of the practical application of BTO@PCNFs interlayer, full cells were assembled for analysis. BTO@PCNFs‐Zn and Bare Zn were used as the anodes respectively, the electrolyte was 2 m ZnSO_4_/0.2 m MnSO_4_, and α‐MnO_2_ was used as the cathodes. The synthesis of α‐MnO_2_ was demonstrated by XRD pattern and FESEM images (Figure S11, Supporting Information). As shown in **Figure** **7**a, the Zn anode was modified by covering the Zn foil with a BTO@PCNFs membrane. The difference in reaction kinetics between the addition of BTO@PCNFs interlayer and Bare Zn was analyzed by analyzing the CV curves (Figure S12, Supporting Information) for the initial four cycles of the full cell. Comparing the CV curves of the second cycle (Figure 7b), the BTO@PCNFs‐Zn//α‐MnO_2_ full cell exhibited higher current intensity and lower voltage polarization, demonstrating that Bare Zn had stronger reaction kinetics as well as electrochemical properties after interfacial modification. Then rate performance tests and cycle stability tests were performed on the full cells. As shown in Figure 7c, the capacity of BTO@PCNFs‐Zn//α‐MnO_2_ full cell was higher than that of Bare Zn//α‐MnO_2_ full cell at different current densities. At 0.2 A g^−1^, the BTO@PCNFs‐Zn//α‐MnO_2_ full cell had better cycling stability and higher capacity retention than Bare Zn//α‐MnO_2_ full cell after 500 cycles (Figure 7d). Furthermore, to explore the potential application of BTO@PCNFs‐Zn electrodes, full cells with BTO@PCNFs‐Zn and Bare Zn as the anodes respectively and VO_2_ as the cathodes were assembled (Figure S13, Supporting Information). It is worth noted that the electrolyte used in this study was 3 m Zn(CF_3_SO_3_)_2_. By analyzing CV curves, BTO@PCNFs‐Zn//VO_2_ full cells had lower polarization voltage and fast kinetics (Figure S14, Supporting Information). Meanwhile, EIS tests also showed that BTO@PCNFs‐Zn//VO_2_ full cells had lower charge transfer resistance (Figure S15, Supporting Information). Impressively, the BTO@PCNFs‐Zn//VO_2_ full cell exhibited outstanding cycling stability and high CE (Figure 7e). At 2 A g^−1^, even after 1000 cycles, the capacity retention rate of the BTO@PCNFs‐Zn//VO_2_ full cell was still 96.6%, while that of the Bare Zn full cell was only 9.6%, demonstrating the excellent stability and reversibility of the BTO@PCNFs‐Zn electrode. This excellent electrochemical performance was owed to the ability of the BTO@PCNFs interlayer to effectively inhibit Zn dendrite growth, inhibit corrosion and improve ion transport kinetics, resulting in stable cycling of the full cells.

## 3 Conclusions

In summary, a multifunctional interlayer by covering the surface of Zn foil with BTO@PCNFs membrane was constructed, which can effectively improve the electrochemical performance and achieve ultra‐long cycle life. The DFT calculation illustrates that BTO and Zn have good affinity for each other, which can effectively improve ion transport and facilitate the uniform deposition of Zn. COMSOL Multiphysics finite element method (FEM) simulations showed that the BTO@PCNFs membrane greatly improved the uniform distribution of electric field, thus achieving the effect of inhibiting Zn dendrites. Meanwhile, the BTO@PCNFs membrane has excellent anti‐corrosion effect. The BTO@PCNFs membrane interfacial modification enabled Zn//Zn symmetric cells to be stably cycled for over 5250 h at 0.5 mA cm^−2^, 0.5 mAh cm^−2^, while also allowing for long‐term reversible stripping/plating at a ultra‐low overpotential of 14 mV. Even at high current density of 30 mA cm^−2^ (1 mAh cm^−2^), the BTO@PCNFs‐Zn electrode could achieve stable cycling for more than 830 h. In addition, the assembled full cells exhibited excellent rate performance and outstanding capacity retention. This interfacial modification of the interlayer is engineered to achieve excellent electrochemical performance, providing a new reference for the practical application of AZIBs.

## 4 Experimental Section

4.1

4.1.1

##### Material Preparation: Preparation of PCNFs and BTO@PCNFs Membranes

Porous BTO@PCNFs membranes were synthesized by electrospinning technique and subsequent pre‐oxidation, carbonization process. First, 1.4 g of polyacrylonitrile (PAN, *M*w = 150,000, J&K Scientific), 50 mg of F127 (Macklin) and 30 mg of BaTiO_3_ powder (BTO, Aladdin, <100 nm) were added into 15 mL of N, N‐dimethylformamide (DMF, Aladdin, >99.9%) solvent, followed by stirring for 18 h to form a homogeneous spinning solution. The solution was sonicated for 30 min, and the obtained solution was loaded into a 15 mL injection tube with voltage set to −0.5 and 17.5 kV for electrospinning. After spinning, the obtained membranes were peeled off from the aluminum foil and then dried under vacuum at 80 °C overnight. Subsequently, the dried membranes were first stabilized in an air atmosphere at 280 °C for 4 h at a ramp rate of 2 K min^−1^. Then the membranes were carbonized at 800 °C for 6 h under N_2_ atmosphere at a ramp rate of 2 K min^−1^. Finally, BTO@PCNFs membranes were obtained. To investigate the effect of different BTO usage on the cell performance, we also synthesized 10 mg BTO addition and 50 mg BTO addition membranes, which were noted as BTO‐10@PCNFs and BTO‐50@PCNFs, respectively. Except for the different BTO addition, all other conditions were consistent with the above synthesis steps. As a control group, the membranes without the addition of BTO were recorded as PCNFs.

##### Preparation of BTO@PCNFs‐Zn Anodes

The Zn foil (thickness: 50 μm) was cut into discs of 14 mm diameter and the BTO@PCNFs membranes were cut into discs of 16 mm diameter. The BTO@PCNFs membranes were covered on top of the Zn foil to obtain the BTO@PCNF‐Zn anodes.

##### Preparation of α‐MnO_2_ Cathodes

Typically, 3 mmol MnSO_4_·H_2_O and 2 mL 0.5 mol L^−1^ H_2_SO_4_ were dissolved in 60 mL distilled water under magnetic stirring for 10 min and then 20 mL 0.1 mol L^−1^ KMnO_4_ was slowly added into the above solution. The mixture was stirred for 1 h, followed by sonication for 1 h. Finally, the mixture was transferred into a Teflon‐lined autoclave and heated at 120 °C for 12 h. After cooling to room temperature, α‐MnO_2_ was washed with distilled water and dried.

##### Preparation of VO_2_ Cathodes

Vanadium dioxide nanobelts (NBs‐VO_2_) were synthesized by the solvent‐thermal alcohol reduction reaction of vanadium pentoxide Vanadium pentoxide (4 mmol) was dispersed in a 50 mL water/ethanol (v/v: 3:2) mixture with vigorous stirring, transferred to a 100 mL autoclave and stored in an oven at 200 °C for 6 h. At the end of the reaction, the reaction was thoroughly washed several times with distilled water and ethanol and dried at 80 °C for 24 h.

##### Materials Characterization

The morphological characteristics of the materials were characterized by scanning electron microscopy (SEM, S‐3400N), field emission scanning electron microscopy (FESEM, JSM‐7800F) equipped with an energy‐dispersive spectrometer (EDS, TEAM Octane Plus), and transmission electron microscopy (TEM, JEM‐2100) equipped with an EDS (EDS, X‐Max80). The phases and structures of the samples were characterized by X‐ray diffraction (XRD, Empyrean). Wettability of zinc anodes was tested by contact angle measurement instruments. The dendrite growth of the zinc anode was observed in real time using in situ optical microscopy. The N_2_ adsorption/desorption isotherms and the corresponding pore size distribution were examined by nitrogen adsorption‐desorption tests. The mechanical properties of BTO@PCNFs interlayer were examined using tensile test (CMT6103).

##### Electrochemical Measurements

Electrochemical tests were performed by assembling CR2032 coin cells. For Zn//Zn symmetric cells and Zn//Cu asymmetric cells, a glass fiber diaphragm (Whatman, GF/D) and a 2 m ZnSO_4_ electrolyte were used. The voltage cutoff for the coulombic efficiency (CE) test in Zn//Cu cells at a current density of 1 mA cm^−2^ was 1 V. The voltage window values for the linear sweep voltammetry (LSV) test were between −0.3 and 0.3 V with respect to the open circuit voltage. For Zn//α‐MnO_2_ cells, a glass fiber diaphragm (Whatman, GF/D) and a 2 m ZnSO_4_/0.2 m MnSO_4_ electrolyte were used. The above electrolytes were added at a volume of 120 μL. The mass ratio of α‐MnO_2_ nanorods, Ketjenblack and PVDF was 7:2:1, and NMP was used as the solvent. The average loading of α‐MnO_2_ was 1.310 mg cm^−2^. Cyclic voltammetry (CV, 0.1 mV s^−1^, 1≈1.8 V) were tested using an electrochemical workstation (VSP‐300). For the Zn//VO_2_ full cells, the electrolyte was 3 m Zn(CF_3_SO_3_)_2_, the mass ratio of VO_2_, Ketjenblack and PVDF was 7:2:1, and the average loading of VO_2_ was 1.414 mg cm^−2^. The sweep rate of the cyclic voltammetry curve was 0.1 mV s^−1^, and the voltage range was 0.3≈1.4 V. Galvanostatic charge/discharge (GCD) measurements were performed by a battery test system (Neware). Electrochemical impedance spectroscopy (EIS) was measured in the frequency range of 100 kHz to 0.01 Hz with an AC voltage amplitude of 5 mV (VSP‐300). Zn//Zn symmetric cells were assembled at room temperature to determine the zinc ion mobility number (t_Zn2+_), calculated as

(1) $$t_{{\text{zn}}^{2+}}=\frac{R_{\text{cell}}}{R_{\text{DC}}}$$

where *R*_cell_ is the total cell resistance before polarization, *R*_DC_ = *V*_DC_/*I*_DC_, *V*_DC_ for the applied potential (10 mV) and *I*_DC_ for the steady current.^[^ ^71^ ^]^

##### Calculation Method

Density functional theory (DFT) calculations were performed using the quantum espresso (QE) based on the pseudopotential plane wave (PPW) method.^[^ ^74^, ^75^ ^]^ The Perdew–‐Bueke–Ernzerhof (PBE) functional was used to describe exchange‐correlation effects of electrons.^[^ ^76^ ^]^ We have chosen the projected augmented wave (PAW) potentials to describe the ionic cores and take valence electrons into account using a plane wave basis set with a kinetic energy cutoff of 500 eV.^[^ ^77^ ^]^ The slab model with 15 Å thick vacuum layer added along the z direction was constructed. The model structures were fully optimized for the cell and ionic degrees of freedom using the convergence criteria of 10^−5^ eV for electronic energy and 10^−2^ eV Å^−1^ for the forces on each atom. The electronic adsorption energies were calculated as

(2) $$E_{\text{ads(mol)}}=E_{\text{slap + mol}}-E_{\text{mol}}-E_{\text{slap}}$$

where *E*_slab_, *E*_mol_ and *E*_slab + mol_ were the energies of slab without the adsorbed molecules, the energy of isolated molecule and the energy of the slab with the molecule adsorbed. According to this definition, a more negative adsoprtion energy denotes a more favorable adsorption.

## Highlights

1. DFT calculations showed that BTO nanoparticles had high zincophilicity, which were favorable for capturing Zn^2+^ and accelerating Zn^2+^ transport as well as inducing uniform Zn deposition.

2. The high dielectric coefficient of BTO can be induced to polarize under the influence of external electric field to form internal electric field; the conductive three‐dimensional (3D) carbon skeleton can further homogenize the electric field. FEM showed that the BTO@PCNFs membrane could homogenize the electric field distribution.

3. The BTO@PCNFs membrane with negative surface charges can shield the anion (SO_4_^2−^) and inhibit the side reaction.

## Conflict of Interest

The authors declare no conflict of interest.

## Supporting information

Supplementary Material

## Data Availability

The data that support the findings of this study are available from the corresponding author upon reasonable request.
