# Supplementary material for: Synergistic Stabilization of Zn Metal Anodes by 3D Carbon Frameworks with Multiple Ion Channels Loaded with Zincophilic BaTiO3 Nanoparticles
Source: Small Sci. 2024 Apr 9;4(6):2400015. doi: 10.1002/smsc.202400015 (PMC11935166; doi:10.1002/smsc.202400015)
Supplement: Supplementary file 1 — Supplementary Material [file SMSC-4-2400015-s001.pdf]

Supplementary Information

Synergistic Stabilization of Zn Metal Anodes by 3D Carbon Frameworks with Multiple Ion Channels Loaded with Zincophilic $BaTiO_3$ Nanoparticles

Chuyi Li [a], Shengyang Jiang [a], Yang Li [a], Yongliang Li [a], Peixin Zhang [a], Chuanxin He [a], Lingna Sun [a,] *, and Hui Ying Yang [b,] *

[a] College of Chemistry and Environmental Engineering, Shenzhen University, Shenzhen 518060, P. R. China

[b] Pillar of Engineering Product Development, Singapore University of Technology and Design 8 Somapah Road, Singapore 487372, Singapore

* Corresponding authors: sunln@szu.edu.cn (Lingna Sun); yanghuiying@sutd.edu.sg (Hui Ying Yang)

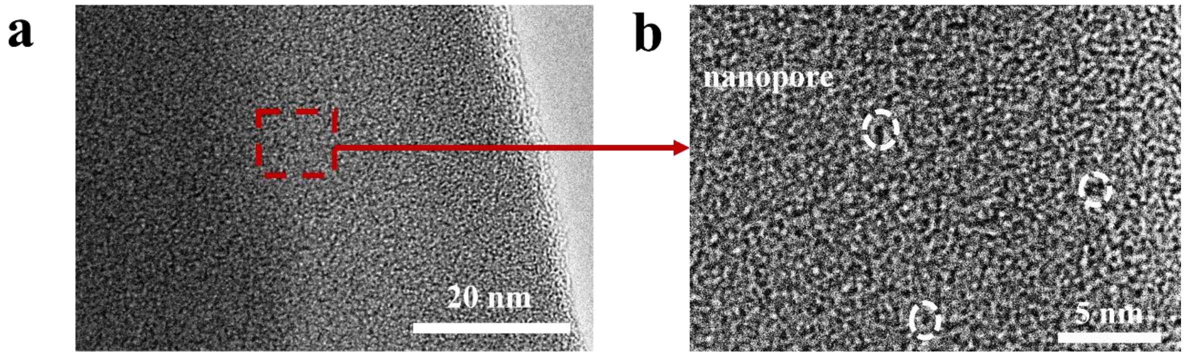

Fig. S1. (a, b) TEM images of BTO@PCNFs membrane.

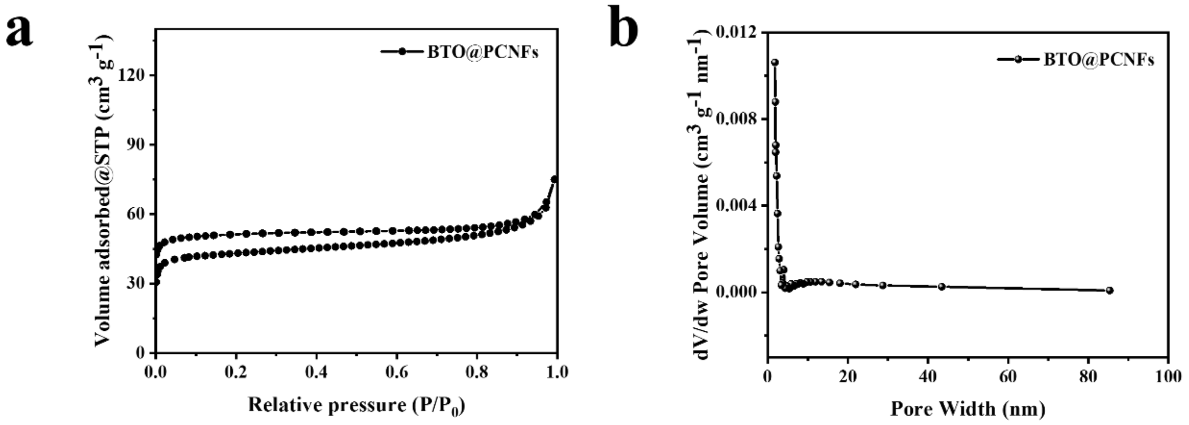

Fig. S2. (a) N_2 adsorption/desorption isotherms; (b) corresponding pore size distribution of the BTO@PCNFs membrane.

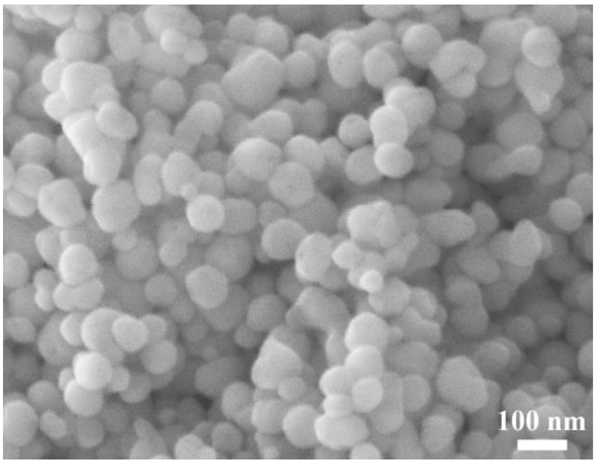

Fig. S3. FESEM image of BTO.

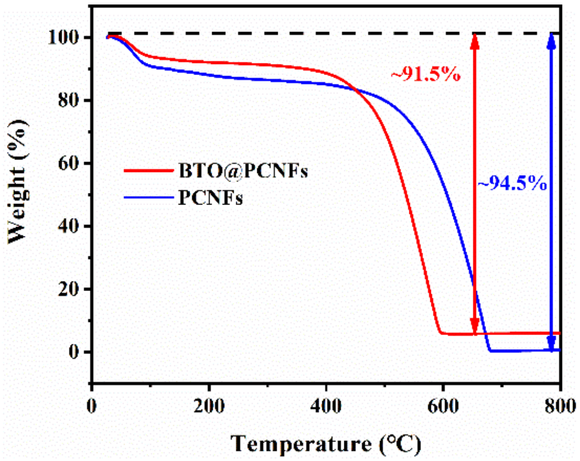

Fig. S4. TG curves of the PCNFs membrane and BTO@PCNFs membrane.

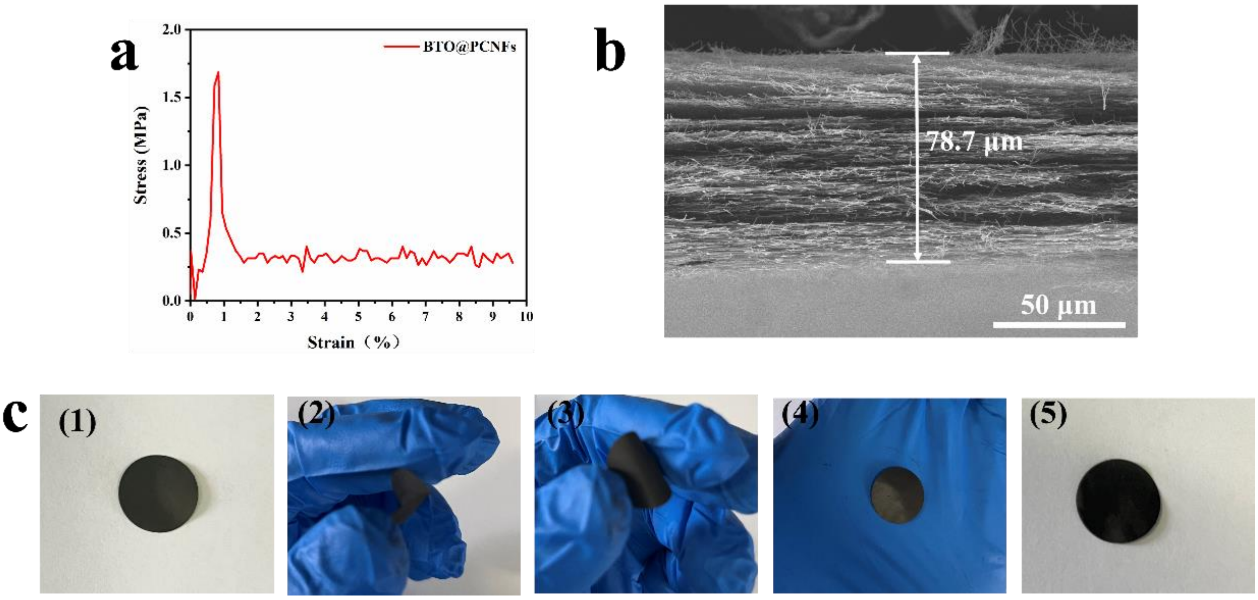

Fig. S5. (a) Tensile test of BTO@PCNFs membrane; (b) Cross-sectional view of BTO@PCNFs membrane; (c) BTO@PCNFs membrane folding tests.

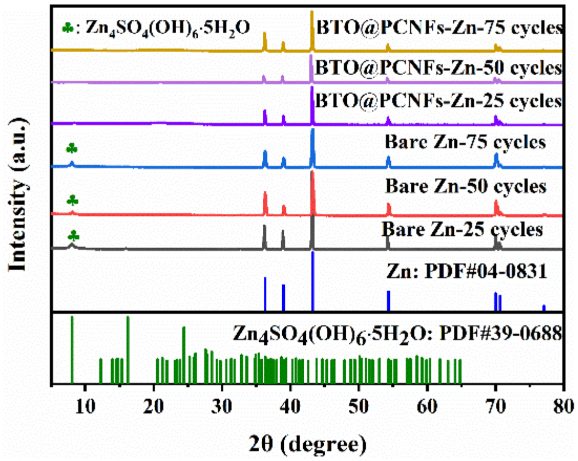

Fig. S6. XRD patterns after cycles. (Test after removal of BTO@PCNFs membrane)

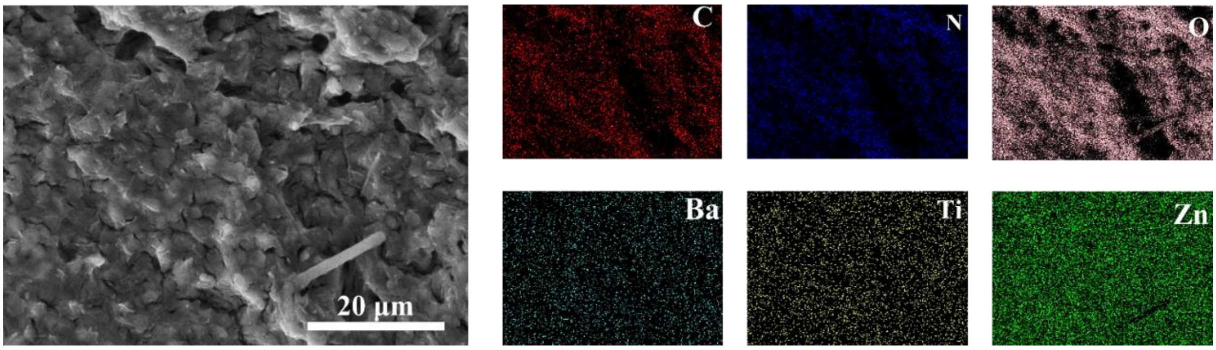

Fig. S7. Elemental mapping images of BTO@PCNFs membrane corresponding to C, N, Ba, Ti, O, Zn after 75 cycles at 5 mA cm^{-2}, 5 mAh cm^{-2}.

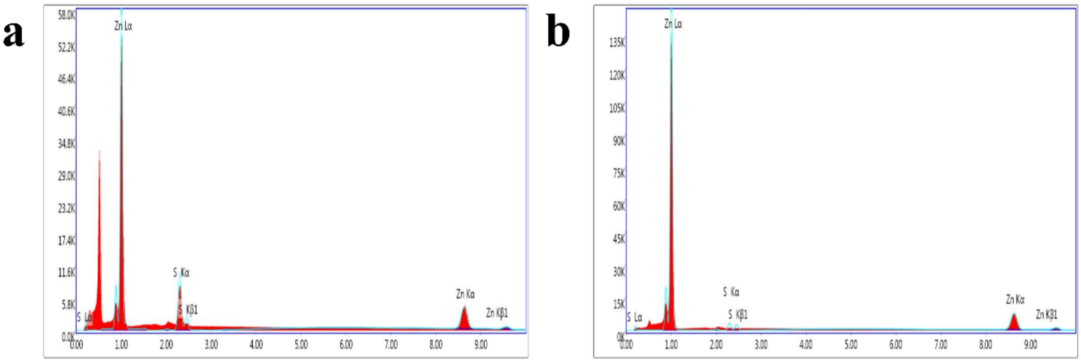

Fig. S8. (a) EDS spectra of bare Zn after 7 days of immersion in 2 M $ZnSO_4$ electrolyte. (b) EDS spectra of Zn foil protected by the addition of BTO@PCNFs membrane after 7 days of immersion in 2 M $ZnSO_4$ electrolyte.

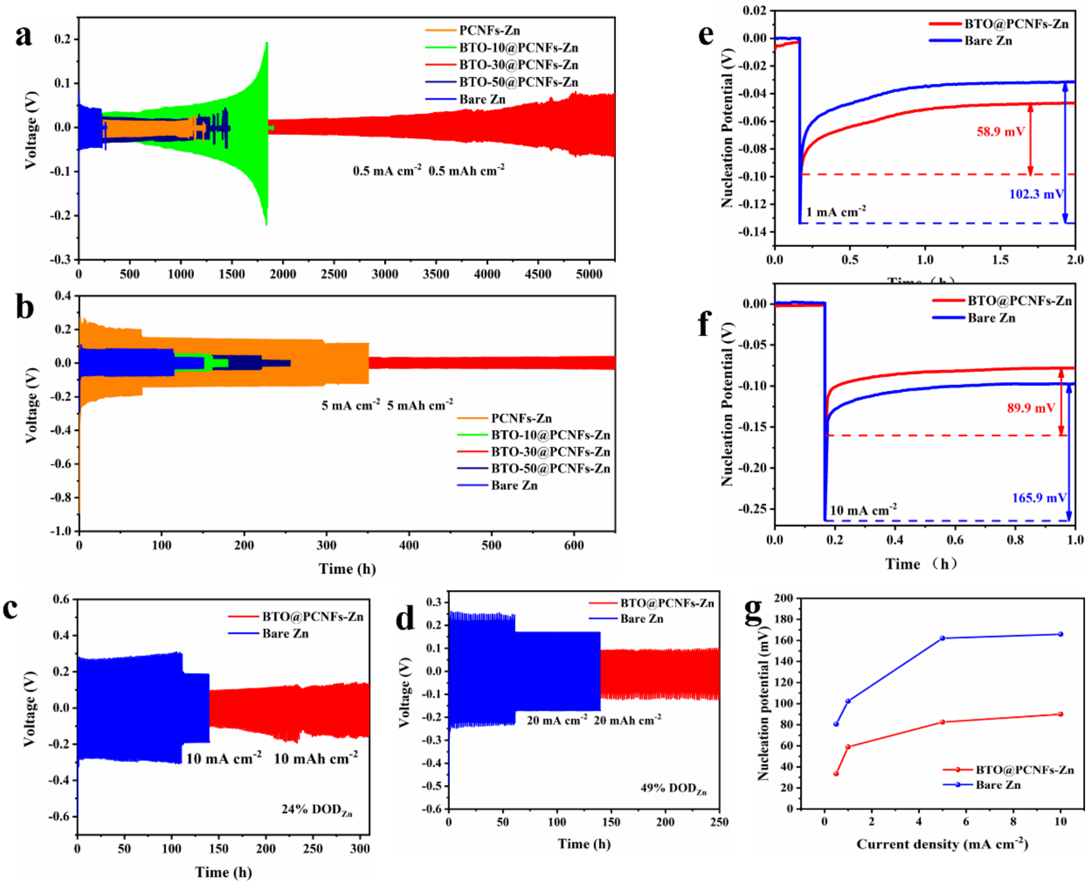

Fig. S9. BTO-10@PCNFs, BTO-30@PCNFs, and BTO-50@PCNFs represent the addition of 10 mg, 30 mg, and 50 mg BTO nanoparticles, respectively. Cycle performance of symmetric cells, (a) at 0.5 mA cm^{-2}, 0.5 mAh cm^{-2}; (b) at 5 mA cm^{-2}, 5 mAh cm^{-2}; (c) at 10 mA cm^{-2}, 10 mAh cm^{-2}; (d) at 10 mA cm^{-2}, 10 mAh cm^{-2}. (e) Nucleation overpotential at 1 mA cm^{-2}. (f) Nucleation overpotential at 10 mA cm^{-2}. (g) Nucleation overpotential at different current density.

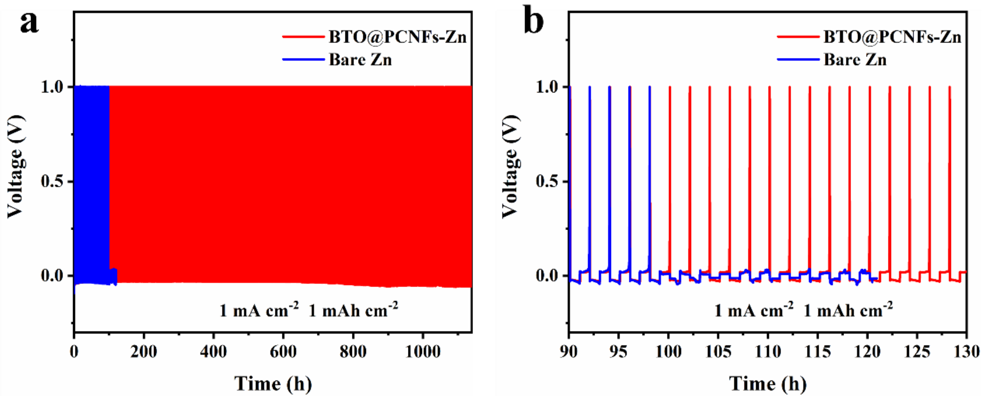

Fig. S10. (a) Galvanostatic Zn plating/stripping behavior of the Zn//Cu asymmetric cell at 1 mA cm^{-2}, 1 mAh cm^{-2}. (b) Partial enlargement of the curve.

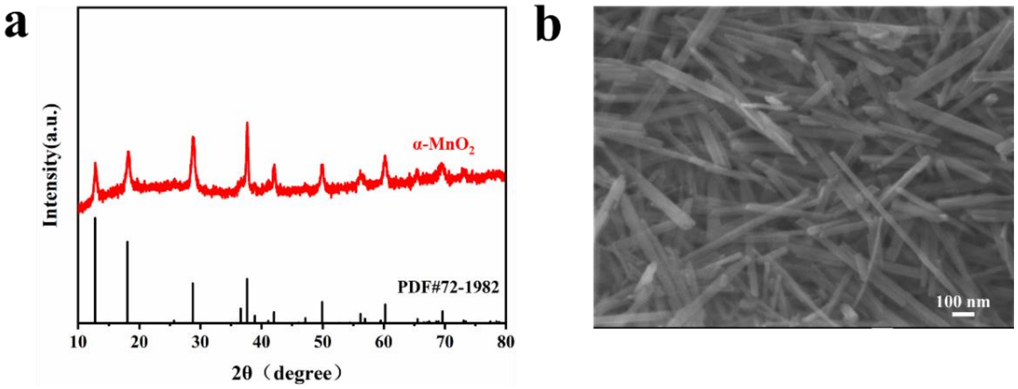

Fig. S11. (a) XRD pattern of α-MnO_2. (b) FESEM image of α-MnO_2

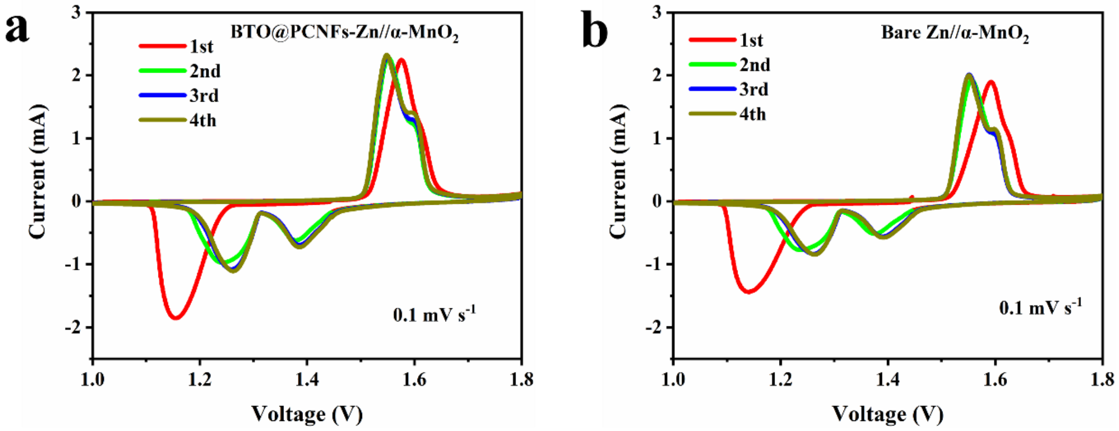

Fig. S12. (a) Initial four-cycle CV curves for BTO@PCNFs-Zn//α-MnO_2. (b) Initial four-cycle CV curves for bare Zn//α-MnO_2.

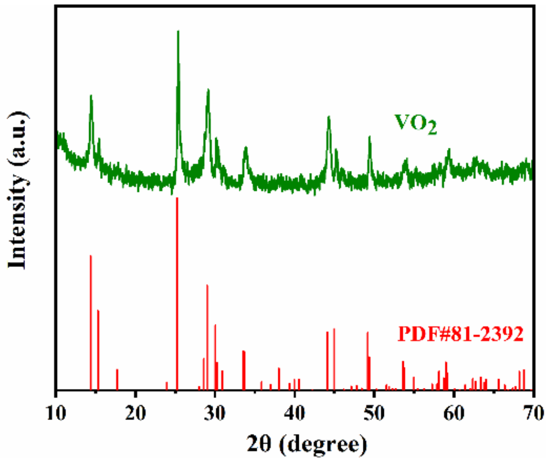

Fig. S13. XRD patten of VO_2.

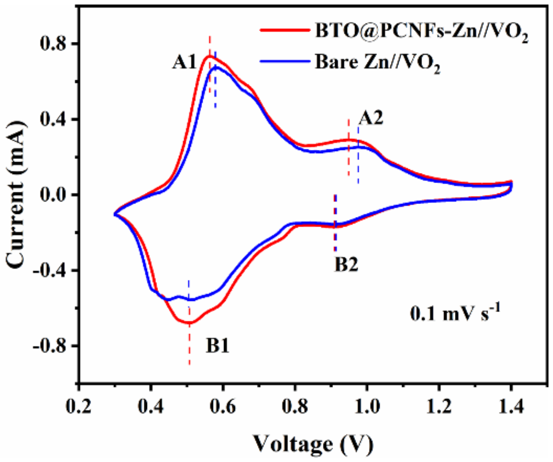

Fig. S14. CV curves of Zn//VO_2 full cells.

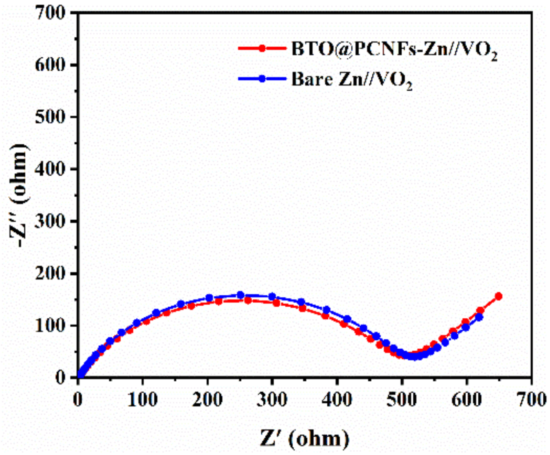

Fig. S15. Nyquist plots of Zn//VO_2 full cells.

Table S1 Comparison of the cycling performance for this work with the similar work.

Electrode	Electrolyte	Current density (mA cm^{-2})	Capacity (mAh cm^{-2})	Life (h)	Cumulative plating capacity (mAh cm^{-2})	Reference
Corona-poled Zn@$BaTiO_3$	1M $ZnSO_4$ + 0.1M $MnSO_4$	1	1	4100	2050	Adv. Energy Mater. 2021, 11, 2100982
		5	2	1450	3625	
		10	5	720	3600	
		20	2	630	6300	
		40	2	158	3160	
Zn@$BaTiO_3$	2 M $ZnSO_4$	1	1	2000	1000	Nano-Micro Lett. 2021, 13, 1
		5	2.5	1500	3750	
Bimodal BTO (90:10) Zn	1 M $ZnSO_4$ + 0.25 M $MnSO_4$	1	1	800	400	ACS Applied Materials & Interfaces 2022， 14， 31
		3	3	250	375	
		5	5	200	500	
BTO/PVT@Zn	2 M $ZnSO_4$	1	1	3000	1500	ACS Energy Lett. 2023, 8, 7,2886–2896
		5	1	950	2375	
DIE separator	2 M $ZnSO_4$	1	1	2500	1250	Adv. Funct. Mater. 2022, 2112936
		10	1	1900	9500	
		10	2.5	1600	8000	
With CNF interlayers	2 M $ZnSO_4$	0.5	0.5	800	200	Chemical Engineering Journal 425 (2021)131862
		1	1	200	100	
		5	1	1200	300	
		5	5	100	250	
**BTO@PCNFs-Zn**	**2 M $ZnSO_4$**	**0.5**	**0.5**	**5250**	**1312.5**	**Our work**
		**5**	**5**	**650**	**1625**	
		**10**	**10**	**310**	**1550**	
		**20**	**1**	**1120**	**11200**	
		**20**	**20**	**250**	**2500**	
		**30**	**1**	**830**	**12450**	

Table S2. Comparison of the cycling performance for this work with recently reported Zn-based symmetric cells based on surface modification strategies.

Electrode	Electrolyte	Current density (mA cm^{-2})	Capacity density (mAh cm^{-2})	Life (h)	Cumulative plating capacity (mAh cm^{-2})	Reference
Zn/Cu-100	1 M $ZnSO_4$	2	1	1000	1000	Energy Storage Materials 56 (2023) 424–431
Cu-MXene-Zn	2 M $ZnSO_4$	10	1	1000	5000	Adv. Funct. Mater.2023, 33, 2213416
		10	10	130	650	
LLP@Treated Zn	2 M $ZnSO_4$	2	1	720	720	ACS Energy Lett. 2023, 8, 8, 3297–3306
		3	0.5	1500	2250	
		10	5	700	3500	
Zn-PA@Zn	2 M $ZnSO_4$	0.5	0.25	2000	500	Energy Environ. Sci., 2022,15, 1872–1881
		1	0.5	1500	750	
		5	2.5	1700	4250	
MXene-mPPy/Zn	2 M $ZnSO_4$	0.2	0.2	2500	250	Adv. Energy Mater.2022, 12, 2103979
		2	4	800	800	
		5	1	1000	2500	
CF-Cu@Zn	2 M $ZnSO_4$	0.5	0.25	2200	550	Adv. Funct. Mater.2022, 32, 2205600
ZnOHF NWs@Zn	2 M $ZnSO_4$	1	1	700	350	Energy Storage Materials 50 (2022) 435–443
		5	1	400	1000	

Zn@Mn	2 M $ZnSO_4$	1	1	4000	2000	Adv.Mater. 2022,34, 2109872
		5	5	85	212.5	
Sn-PCF@Zn	2 M $ZnSO_4$	1	1	750	375	Energy Storage Materials 51 (2022) 259–265
		5	1	700	1750	
		10	5	500	2500	
**BTO@PCNFs-Zn**	**2 M $ZnSO_4$**	**0.5**	**0.5**	**5250**	**1312.5**	**Our work**
		**5**	**5**	**650**	**1625**	
		**10**	**10**	**310**	**1550**	
		**20**	**1**	**1120**	**11200**	
		**20**	**20**	**250**	**2500**	
		**30**	**1**	**830**	**12450**	
